# Clinical Value of lncRNA MEG3 in High-Grade Serous Ovarian Cancer

**DOI:** 10.3390/cancers12040966

**Published:** 2020-04-14

**Authors:** Marianna Buttarelli, Marta De Donato, Giuseppina Raspaglio, Gabriele Babini, Alessandra Ciucci, Enrica Martinelli, Pina Baccaro, Tina Pasciuto, Anna Fagotti, Giovanni Scambia, Daniela Gallo

**Affiliations:** 1 Unit of Translational Medicine for Woman and Child Health, Department of Woman and Child Health and Public Health, Fondazione Policlinico Universitario A. Gemelli, Istituto di Ricovero e Cura a Carattere Scientifico (IRCCS), 00168 Rome, Italy; Marianna.buttarelli1@unicatt.it (M.B.); marta.dedonato@unicatt.it (M.D.D.); giuseppina.raspaglio@unicatt.it (G.R.); Alessandra.ciucci@unicatt.it (A.C.); enrica.martinelli@unicatt.it (E.M.); 2 Department of Life Sciences and Public Health, Section of Gynecology and Obstetrics, Università Cattolica del Sacro Cuore, 00168 Rome, Italy; pina.baccaro@unicatt.it (P.B.); anna.fagotti@policlinicogemelli.it (A.F.); giovanni.scambia@policlinicogemelli.it (G.S.); 3 Department of Woman and Child Health and Public Health, Fondazione Policlinico Universitario A. Gemelli, Istituto di Ricovero e Cura a Carattere Scientifico (IRCCS), 00168 Rome, Italy; gabriele.babini@guest.policlinicogemelli.it (G.B.); pasciuto.tina@gmail.com (T.P.)

**Keywords:** MEG3, lncRNAs, progression-free survival, overall survival, cancer biomarkers, ovary, ovarian cancer cell lines, personalized medicine

## Abstract

Long non-coding RNAs (lncRNAs) are emerging as regulators in cancer development and progression, and aberrant lncRNA profiles have been reported in several cancers. Here, we evaluated the potential of using the maternally expressed gene 3 (MEG3) tissue level as a prognostic marker in high-grade serous ovarian cancer (HGSOC), the most common and deadliest gynecologic malignancy. To the aim of the study, we measured MEG3 transcript levels in 90 pre-treatment peritoneal biopsies. We also investigated MEG3 function in ovarian cancer biology. We found that high MEG3 expression was independently associated with better progression-free (*p* = 0.002) and overall survival (*p* = 0.01). In vitro and in vivo preclinical studies supported a role for MEG3 as a tumor suppressor in HGSOC, possibly through modulation of the phosphatase and tensin homologue (PTEN) network. Overall, results from this study demonstrated that decreased MEG3 is a hallmark for malignancy and tumor progression in HGSOC.

## 1. Introduction

Ovarian cancer is the deadliest gynecologic malignancy. Worldwide, nearly 295,000 women were estimated to have been diagnosed with ovarian cancer and 185,000 to have died from the disease in 2018, with rates varying across the world [1]. The disease typically presents at advanced stage (III–IV) where the 5 year survival is around 30%. Few cases (15%) are diagnosed with localized tumor (stage I), with a 5 year survival rate of 92% [2]. High-grade serous ovarian cancer (HGSOC), also known as high-grade serous carcinoma, is estimated to be 50–60% of all ovarian malignancies, and advanced-stage HGSOC represents nearly a half of all epithelial ovarian cancer [3]. Limited understanding of disease biology and lack of screening tests to diagnose disease early, along with insufficient treatment approaches, represent the main problems concerning HGSOC [4]. Thus, in spite of great progress in some of these aspects, the management of this neoplasm remains a major challenge in gynecologic oncology, and over the last 40 years, long-term HGSOC survival rates have changed very little, with several issues impeding progress in clinical outcome.

Emerging evidence demonstrates that although a large number of RNA species are transcribed from the human genome, protein-coding sequences account for a fraction of total transcripts. The remaining transcripts are non-coding RNAs (ncRNAs) such as small interfering RNAs (siRNAs), microRNAs (miRNAs), and long non-coding RNAs (lncRNAs). lncRNAs comprise a diverse class of RNA transcripts >200 nucleotides in length; they act as key regulators of target gene expression in various biological processes, such as chromatin modification, gene transcription, RNA splicing, and RNA transport and translation [5]. In vitro and in vivo studies have proven that aberrant lncRNA expression may be crucial for the initiation and progression of cancers and that lncRNA-mediated biology might occupy a central place in cancer progression [6,7]. Notably, unlike miRNAs and protein-coding mRNAs, lncRNAs commonly show restricted tissue-specific and cancer-specific expression patterns [8]; moreover, they have lower expression than protein-coding genes [9]. Given this lncRNA tissue specificity, they may therefore be superior biomarkers to many current protein-coding biomarkers [10].

With regard to ovarian cancer, the study of lncRNAs is still in its beginning stages, although the last couple of years have seen a growing number of publications on this topic, which could provide useful evidence for the identification of new biomarkers. Indeed, the altered expression of certain lncRNAs has been reported in ovarian cancer and is associated with clinical-pathological characteristics (reviewed in [11,12]).

The maternally expressed gene 3 (MEG3) is an imprinted gene located on chromosome 14q32.3 in humans that encodes a lncRNA-MEG3 expressed in many tissues, including the ovaries [13]. Recent studies have shown that expression of MEG3 is lost in multiple types of tumors, with lines of evidence strongly suggesting that MEG3 functions as a novel lncRNA tumor suppressor [13,14]. Multiple mechanisms may contribute to the loss of MEG3 expression in tumors, including gene deletion, promoter hypermethylation, and hypermethylation of the intergenic differentially methylated region [13]. MEG3 expression is also decreased in ovarian cancer compared to normal tissue, and its promoter is highly methylated [15,16,17]. However, the potential clinical implications of dysregulated MEG3 expression in ovarian cancer is still unclear.

Here, we aimed at elucidating the clinical relevance of MEG3 in a homogenous series of advanced HGSOC patients; then, we investigated its function in ovarian cancer biology. Our results demonstrate that MEG3 may be a useful prognostic marker for HGSOC patients, and its inactivation participates in the regulation of cancer cell development and progression.

## 2. Results

### 2.1. MEG3 Was Found to Be an Independent Favorable Prognostic Factor in Advanced HGSOC

Clinicopathological characteristics of the study cohort are summarized in Table 1. BRCA (BReast CAncer gene) mutational status was available for 51 patients out of 90, and therefore it was not included in survival analyses.

**Figure 1:**
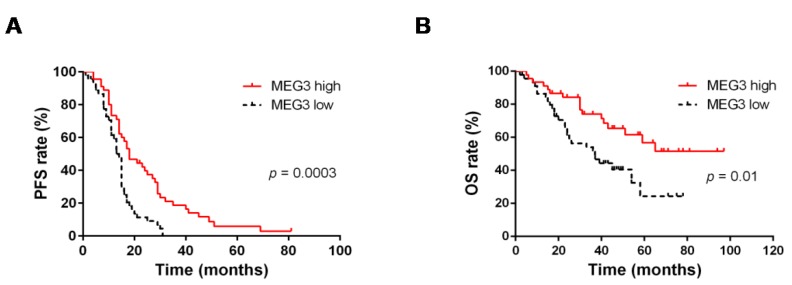
Kaplan–Meier survival curves for the probability of (**A**) progression-free survival (PFS), and (**B**) overall survival (OS), according to expression of maternally expressed gene 3 (MEG3) in advanced high-grade serous ovarian cancer (HGSOC) patients. MEG3 expression levels were converted into discrete variables by dividing the available samples (population size *n* = 90) into high and low expression, over or under the cut-off (i.e., median expression level). Results of log-rank tests are shown.

To evaluate the prognostic potential of MEG3 in HGSOC, we assessed its expression in 90 peritoneal biopsies and correlated results with progression-free survival (PFS) and overall survival (OS) in univariate and multivariate analyses, adjusted for clinicopathological parameters. Results obtained in univariate log-rank test showed that MEG3 correlated with PFS (*p* = 0.0003, Figure 1A and Table 2). High and low MEG3 cases showed median PFS values of 18 and 14 months, respectively. Notably, in multivariate Cox regression analysis, after adjusting for residual tumor after surgery, MEG3 was identified as an independent predictor of PFS (*p* = 0.002, Table 2). Univariate log-rank analysis also identified MEG3 as being significantly associated with OS (*p* = 0.01, Figure 1B and Table 3). Although a median of 37 months was found in low-MEG3 cases, median OS was not reached in patients with high MEG3 levels. In multivariate Cox regression analysis, after adjusting for age, MEG3 was again identified as an independent predictor of OS (*p* = 0.01, Table 3). Finally, Fisher’s test showed an association between MEG3 expression and sensitivity to first line chemotherapy (*p* = 0.05, Table 4). No significant association with other clinicopathological characteristics of the disease was observed (Table 4).

### 2.2. MEG3 Expression in HGSOC Cell Lines

**Figure 2:**
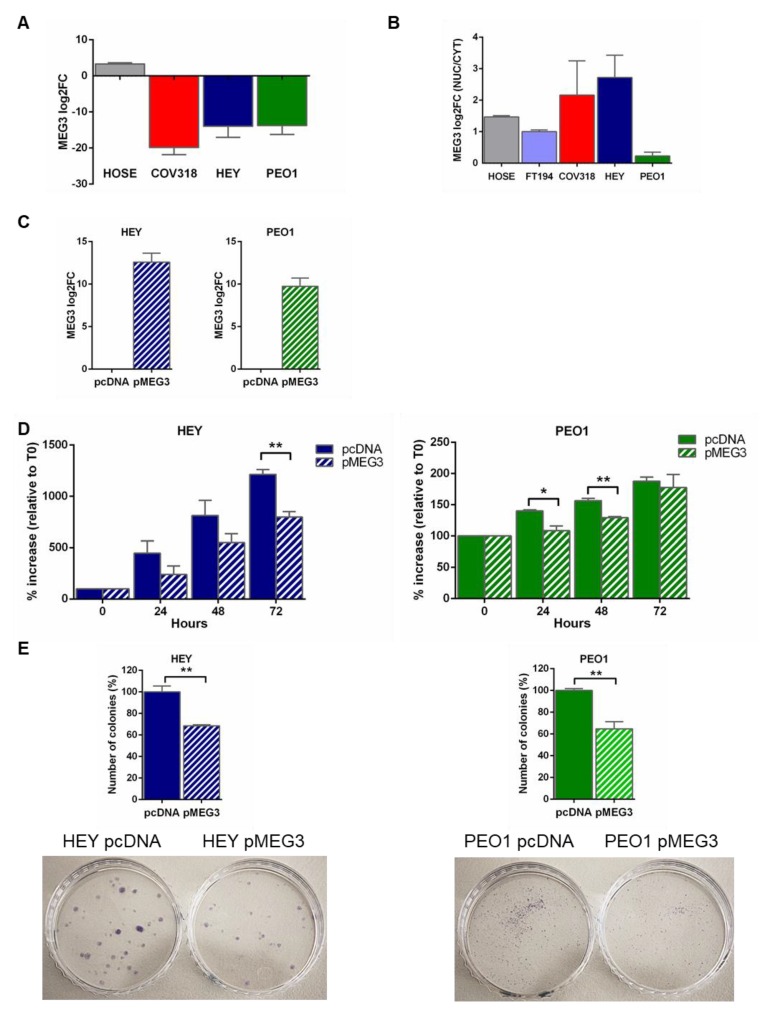
MEG3 modulation affected proliferation and clonogenic capability of high-grade serous ovarian cancer (HGSOC) cells. (**A**) Relative MEG3 expression and (**B**) relative MEG3 subcellular localization assessed by RT-qPCR analysis in a panel of HGSOC cell lines as well as in HOSE (Human ovarian surface epithelial cells) and FT194 (Fallopian Tube epithelial cells). Data from total RNA are presented as Log2 fold change (Log2FC) values calculated with the ΔΔCt method, using FT194 as a reference sample. Relative MEG3 expression in nucleus vs. cytoplasm is expressed as Log2FC values calculated with the ΔCt method and compared to FT194 cells; positive values represent nuclear enrichment. (**C**) Relative MEG3 expression assessed by RT-qPCR analysis in HEY and PEO1 cells (HGSOC cell lines) transiently transfected with pMEG3 (MEG3 expression plasmid) and with pcDNA (empty vector). Data are presented as Log2 fold change (Log2FC) values calculated with the ΔΔCt method, using pcDNA as reference sample. (**D**) Bar chart representing proliferation assay for HEY-pMEG3 and PEO1-pMEG3 cells compared to respective control cells. Viable cells were counted at 24, 48, and 72 h from transfection. The mean cell proliferation at time x (Tx) was expressed as average percentage increase relative to T = 0 h (T0). (**E**) Clonogenic assay. Bar charts represent differences of clonogenic capability in MEG3 overexpressing cells with respect to control cells and representative pictures of clonogenic assays. For all experiments, bars and error bars refer to mean and SEM (standard error of the mean) of three experiments. To establish statistically significant differences, unpaired *t*-test was carried out: * *p* < 0.05; ** *p* < 0.01.

As a first step in the investigation of the role of MEG3 in cancer development, we assessed the expression and cellular localization of the transcript in a panel of cell lines selected among those considered as really representative of HGSOC [18,19,20], and in normal epithelial HOSE (Human ovarian surface epithelial) and FT194 (Fallopian tube epithelial) cells for comparison. Interestingly, results demonstrated that MEG3 expression was considerably downregulated in HGSOC cells compared to FT194 (as well as to HOSE cells) (Figure 2A). Our results also showed a preponderating nuclear localization of the transcript both in normal epithelial cells and in HGSOC cell lines, although at a different extent in the different cell lines (Figure 2B).

### 2.3. MEG3 Regulated the Proliferation of HGSOC Cells

Thereafter, in order to assess the effect of MEG3 on tumor cell proliferation and clonogenic capability, we transfected HEY and PEO1 cells (HGSOC cell lines) with pMEG3 (MEG3 expression plasmid) to transiently over-express the transcript. After 24 h from transfection, the expression of MEG3 in cells was assessed by RT-qPCR. Results demonstrated that MEG3 level in the pMEG3 cells was considerably increased compared to the control (Figure 2C), thus confirming a successful transfection. After exogenous MEG3 overexpression, we observed a significant decrease in cell proliferation at 72 h in HEY cells (*p* = 0.004 vs. control), and at both 24 and 48 h in PEO1 (*p* = 0.02 and *p* = 0.003 vs. control, respectively) (Figure 2D). Similarly, clonogenic assays revealed a low capability of colony formation in HEY and PEO1 cells overexpressing MEG3 with respect to control cells (*p =* 0.005 and *p* = 0.007, respectively) (Figure 2E).

### 2.4. MEG3 Overexpression Inhibited Cell Migration and Invasion of HGSOC Cells

**Figure 3:**
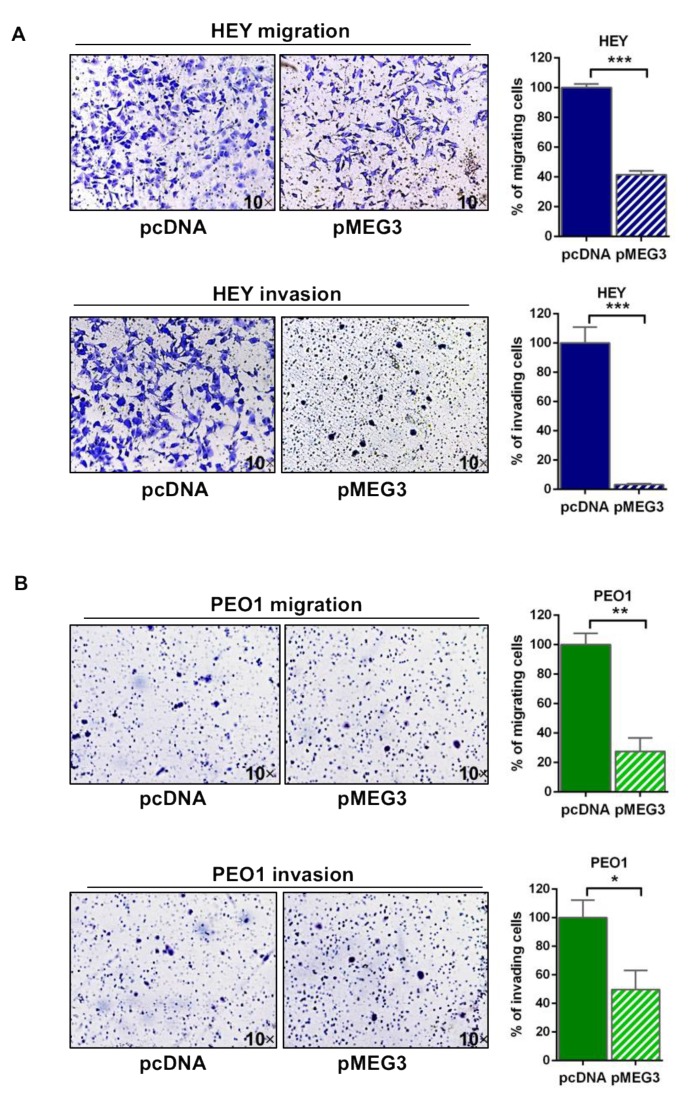
MEG3 overexpression inhibited cell migration and invasion of high-grade serous ovarian cancer (HGSOC) cells. Transwell migration and invasion assays in (**A**) HEY and (**B**) PEO1 cells transfected with pMEG3 and empty vector pcDNA as control and representative pictures of HEY and PEO1 transwell migration and invasion assays. Values are expressed as percentage of migrating or invading cells relative to control cells. Bars and error bars refer to mean and SEM of three experiments. To establish statistically significant differences, unpaired *t*-test was carried out: * *p* < 0.05; ** *p* < 0.01; *** *p* < 0.001. Magnification: 10×.

By transwell migration and invasion assays, we then evaluated the migration and invasion abilities of HEY and PEO1 cells transfected with pMEG3 or empty vector (Figure 3A,B). Notably, in line with recent literature data, PEO1 exhibited relative low migration and invasion abilities [21]. Results obtained showed a significant reduction of migration ability in MEG3-overexpressing tumor cells compared to the control (*p* < 0.001 and *p* = 0.004 for HEY and PEO1, respectively). Analysis of cell invasion corroborated these data, showing a reduced spreading of MEG3-overexpressing cells compared to empty vector (*p* < 0.001 and *p* = 0.04 for HEY and PEO1, respectively).

### 2.5. MEG3 Overexpression Inhibited Spheroid Growth in Extracellular Matrix

**Figure 4:**
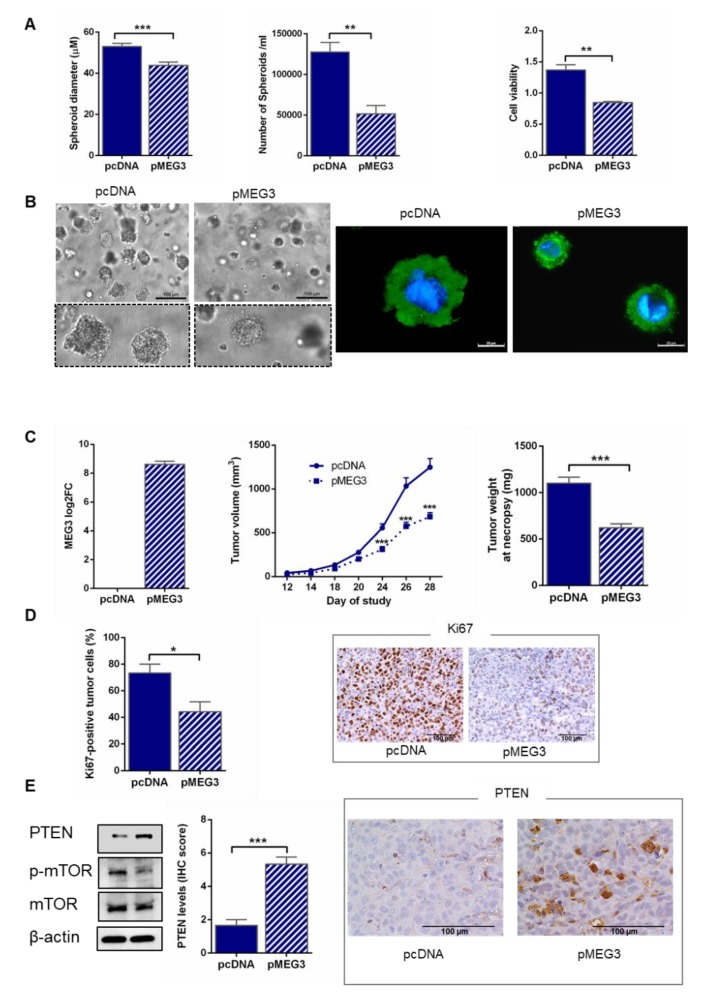
MEG3 overexpression inhibited high-grade serous ovarian cancer (HGSOC) spheroid growth in extracellular matrix and tumor growth in mice. (**A**) Spheroid assays results using transfected HEY pcDNA and pMEG3 cells after 10 days of culture. Left panel: bar chart showing spheroid size (µm), measured by Leica Application Suite (LAS) analysis. Twenty spheroids were analyzed for each data point. Middle panel: bar chart showing spheroid number assessed by counting spheres in the Neubauer chamber. Right panel: bar chart showing cell viability, measured by Cell Counting Kit 8 (CCK-8) assay, after 10 days of cell culture. Bars and error bars refer to mean and SEM of two experiments. (**B**) Left panel: representative bright field microscopy (20×; scale bars, 100 µm) and zoomed-in images of transfected HEY pcDNA and pMEG3 spheroids released from the hydrogel. Right panel: representative immunofluorescence (100×; scale bars, 20 µm) images of transfected HEY pcDNA and pMEG3 spheroids released from the hydrogel. For immunofluorescence, the images show the merged signal of Alexa Fluor 488 β-actin mouse monoclonal antibody (green) and DAPI (4′,6-diamidino-2-phenylindole) (blue). (**C**) Tumor burden was significantly decreased in female athymic mice inoculated with stably transfected HEY pMEG3 compared to pcDNA (a suspension of 2.0 × 10^6^ cells was injected subcutaneously in the right flank of each animal). Left panel: relative MEG3 expression assessed by RT-qPCR analysis in stably transfected HEY pcDNA and pMEG3 cells at the time of inoculation in mice. Data from total RNA are presented as Log2 fold change (Log2FC) values calculated with the ΔΔCt method, using pcDNA cells as reference sample. Middle/right panels: tumor growth curve and tumor wet weight. Values are means and SEM, *n* = 12 mice per group. (**D**) Bar charts and representative immunohistochemical pictures showing expression of Ki67 in tumors from HEY pcDNA and pMEG3 groups. Values are means and SEM, *n* = 6 tumors per group. (**E**) Left panel: Western blot analysis of stably transfected HEY pcDNA and pMEG3 cells at the time of inoculation in mice. Middle/right panels: bar charts and representative immunohistochemical pictures showing phosphatase and tensin homologue (PTEN) expression in tumors from HEY pcDNA and pMEG3 groups (40×; scale bars, 100 µm). Values are means and SEM, *n* = 6 tumors per group. To establish statistically significant differences, unpaired *t*-test was carried out: * *p* < 0.05; ** *p* < 0.01; *** *p* < 0.001.

Multicellular tumor spheroid systems better recapitulate in vivo growth conditions, thus allowing more faithful reproduction of broader aspects of tumor biology. Importantly, the formation of tumor spheroids is a key step in the metastatic process of ovarian cancer. Therefore, we developed in vitro 3D culture from HEY pcDNA (empty vector) and pMEG3 cells, obtaining small spheroids with diameters of about 50 µm. Interestingly, expression of MEG3 significantly reduced the extent of cell aggregation, as demonstrated by the decreased spheroid size (*p* < 0.001, Figure 4A,B). Moreover, a lower number (*p* = 0.008) and a reduced viability of cells (*p* = 0.004) were observed in MEG3-overexpressing HEY spheroids compared to HEY pcDNA (Figure 4A,B). These results show that MEG3 strongly represses metastatic tumor potential in HGSOC cells.

### 2.6. MEG3 Overexpression Inhibited the Growth of HGSOC in Mice

Finally, to confirm the effect of MEG3 on HGSOC cells in vivo, we subcutaneously injected stably transfected HEY pcDNA and pMEG3 into athymic Balb/C mice. Before mice dosing, we assessed by RT-qPCR the expression of MEG3 in cells; results demonstrated that MEG3 level in the pMEG3 cells was considerably increased compared to the control (Figure 4C), thus confirming a successful transfection. Tumor growth was significantly suppressed in pMEG3 mice compared with pcDNA group (*p* < 0.001, Figure 4C); accordingly, tumor weight at the end of the study was lower in MEG3-overexpressing xenografts compared to the control group (*p* < 0.001, Figure 4C). The proliferation index Ki67 was significantly lower in xenografts from pMEG3 mice than in pcDNA group (*p* = 0.02, Figure 4D).

### 2.7. MEG3 Overexpression Upregulated PTEN

Due to recent studies showing a role for phosphatase and tensin homologue (PTEN) in mediating MEG3 tumor suppressor activity in ovarian cancer cells [22], we evaluated PTEN expression in stably transfected HEY pcDNA and pMEG3 cells. Western blot analysis showed a relevant protein increase in pMEG3 overexpressing cells compared to controls (Figure 4E; Figure S1: Uncropped blots of Figure 4E; Table S2: Densitometric analysis of Figure 4E); notably, this modulation was supported by the concomitant decrease in mTOR (Mammalian target of rapamycin) phosphorylation (Figure 4E; Figure S1: Uncropped blots of Figure 4E; Table S2: Densitometric analysis of Figure 4E), in line with literature findings of key pathways regulated by PTEN [23]. The regulation of PTEN by MEG3 was then confirmed in immunohistochemical analysis of mouse xenografts, showing that the protein was significantly up-regulated in pMEG3-overexpressing tumor compared to controls (*p <* 0.001, Figure 4E). Taken together, these findings demonstrate that MEG3 inhibits malignant progression of HGSOC cells in vitro and in vivo, at least partially, through the modulation of the PTEN network.

## 3. Discussion

Evidence is increasing that lncRNAs play a key role in tumorigenesis and tumor progression, and dysregulated lncRNA profiles have been reported in several malignancies. Here, we aimed to explore the role of MEG3 as a prognostic biomarker in a homogenous series of advanced HGSOC patients, as well as to investigate its function in cancer development. Results obtained showed that MEG3 is a powerful prognostic biomarker of clinical utility and its level might affect the efficacy of first line therapy in advanced HGSOC patients. Preclinical data also show promise for personalized treatment approach, acting on MEG3-mediated pathways.

Previous studies on the clinical relevance of MEG3 in ovarian cancer have produced conflicting results. Filippov-Levy and colleagues [24] reported a lack of association between effusion levels of MEG3 and prognosis in a cohort of HGSOC patients. On the other hand, results from a more recent study actually support an unfavorable prognostic role for underexpressed MEG3 in patients with OC [25], in line with our data. The discrepancy among different reports may be due, at least partially, to differences in the patient cohort and/or in the specimens examined. It is worthy to note, however, that according to the Cancer LncRNA Census (CLC), a compilation of 122 GENCODE lncRNAs with causal roles in cancer phenotypes, MEG3 is among the top-ranked lncRNAs, on the basis of available data relative to its role as tumor suppressor [26].

To characterize the specific functions of MEG3 in HGSOC, we firstly measured its abundance in a panel of HGSOC cell lines compared to HOSE and FT194, showing that MEG3 downregulation could be a common step in cancer development, independently of cell of origin. In addition, because lncRNA subcellular localization may be informative regarding their biological function, we also assessed subcellular localization of MEG3 in our panel of cell lines. We found that, as was the case in HOSE and FT194 control cell lines, residual MEG3 in HGSOC cells still keep a preponderant nuclear localization. Our data on nuclear MEG3 enrichment are consistent with literature data [27], supporting a role for this transcript in transcriptional regulation that needs, however, to be fully elucidated in the context of HGSOC. Overall, our in vitro and in vivo experimental studies demonstrated that exogenous MEG3 expression is able to revert the malignant phenotype in HGSOC. Indeed, in line with recent literature data [22,28], we proved that over-expression of MEG3 inhibited cell proliferation, plate colony formation, migration, and invasion ability, as well as spheroid formation and tumor growth in mice.

With regard to mechanism of action, the bulk of evidence indicates that MEG3 acts as a tumor suppressor through the accumulation of p53 protein and successive activation of its downstream target genes [13]; this mechanism, however, cannot be relevant in the context of HGSOC, characterized by mutation in the TP53 gene [29]. On the other hand, recent studies have implicated MEG3 in the regulation of different oncogenic and tumor-suppressive gene networks, possibly through an activity as a molecular decoy for cancer-associated microRNAs [28,30]. Indeed, one of the most interesting mechanisms of action reported for MEG3 is the positive regulation of PTEN expression in ovarian cancer cells [22]. Although driver pathways for this modulation have not been entirely clarified, one possibility is that MEG3 could act as competitive endogenous RNA for miR-214 [31] that, in turn, negatively regulates PTEN protein expression [32]. Of note, miR-214 has also been implicated in ovarian cancer, with reports demonstrating that it may induce cisplatin resistance in ovarian cancer cells [31]. In line with these previous findings, our preliminary mechanistic investigation actually supported the possibility that PTEN could be a downstream target of MEG3. Notably, evidence exists that PTEN expression is lost in the majority of HGSOC cases through a combination of DNA alterations and transcriptional or post-transcriptional modulations; moreover, PTEN loss has an unfavorable prognostic value in HGSOC, and it is associated with chemoresistance [33,34]. The loss of PTEN functions impairs different key biological processes, ranging from inhibition of cell growth, proliferation, and migration, to promotion of apoptosis and tumor suppressor activity [23], findings that closely resemble our preclinical data. Relevant to our in vitro findings is also the observation that loss of PTEN in fallopian tube epithelium results in multicellular tumor spheroid formation and metastasis to ovary [35].

Overall, our results provide evidence that MEG3 may serve as a powerful independent prognostic factor for both PFS and OS in advanced HGSOC patients, although future studies are required to validate this finding. Our data also elucidate an important key role of MEG3 in the molecular biology of HGSOC, implicating a novel target for diagnosis, prognosis, and future targeted therapies.

## 4. Materials and Methods

### 4.1. Study Design and Patients

Tissue samples used in the study were derived from the SCORPION clinical trial. This was a superiority, randomized phase III trial (no. NCT01461850) investigating whether neoadjuvant chemotherapy (NACT) followed by surgery is superior to primary surgery in terms of progression-free survival (PFS) in advanced epithelial ovarian cancer (EOC) patients endowed with high tumor load [36,37]. Patients received platinum-based chemotherapy. Introduction of bevacizumab in combination with standard chemotherapy, and as maintenance therapy, was allowed in both arms, since its approval in Italy (January, 2014). Response to chemotherapy and progression were defined according to Response Evaluation Criteria in Solid Tumors (RECIST) and Gynecologic Cancer Intergroup (GCIG) criteria [38,39]. To define chemosensitivity, the common definition of platinum resistance was used, identifying patients that relapsed 6 months or more after prior platinum-containing chemotherapy as “sensitive”, and patients that relapsed less than 6 months after chemotherapy was stopped, or that progressed during therapy, as “resistant” [40].

Peritoneal biopsies taken at staging laparoscopy from both primary debulking surgery (PDS) and NACT/interval debulking surgery (IDS) arms were collected before any treatment. MEG3 analysis was carried out on formalin-fixed paraffin embedded (FFPE) tissues. We decided to use FFPE samples because any biomarker developed from these can be more readily translated into clinical practice. After a review of the available paraffin blocks, RNA extraction, quality control, and profiling by RT-qPCR, we considered 90 pre-treatment HGSOC tissue samples as eligible for data analysis. Follow-up data were available for all 90 patients (median follow-up, 50 months, 95% CI: 41–71). During the follow-up period, progression and death of disease were observed in 86 and 44 patients, respectively.

The study complied with the Ethical Principles for Medical Research Involving Human Subjects according to the World Medical Association Declaration of Helsinki, and was approved by the Committee of “Fondazione Policlinico Universitario Agostino Gemelli IRCCS-Università Cattolica del Sacro Cuore”, Roma (Protocol no. 19309/18 - ID:2048). Patients signed a written consent form for data and sample collection and for the use of personal records for health research. All data were managed using anonymous numerical codes.

### 4.2. RNA Extraction from FFPE Tumor Tissues and MEG3 Expression Analysis in Cancer Samples

FFPE tumor blocks were evaluated to select regions of invasive carcinoma containing >70% of malignant epithelial cells for macro-dissection. Xylene was added to 5 μm sections of FFPE samples to remove paraffin. RNA was isolated using the RNeasy FFPE Kit (Qiagen, Milan, Italy) according to the manufacturer’s protocol and stored at −80 °C until analyzed. Recovered RNA concentrations and quality were measured using the Nanodrop (Thermo Scientific, Waltham, MA) and Agilent 2100 Bioanalyzer (Agilent Technologies, Santa Clara, CA, USA), respectively. Only samples with a DV200 value (the percentage of fragments > of 200 nucleotides) ≥70% were used.

Quantitative real-time PCR (RT-qPCR) was performed to analyze lncRNA MEG3 expression in cancer samples. A total of 200 ng of RNA was reverse-transcribed, pre-amplified, and subjected to RT-qPCR analysis using CFX Connect Real Time PCR Detection System (Bio-Rad, Hercules, CA, USA), according to the manufacturer’s instructions, as previously described [41]. GAPDH (Glyceraldehyde 3-phosphate dehydrogenase), HNRNPH2 (Heterogeneous Nuclear Ribonucleoprotein H2), RPLP0 (Ribosomal Protein Lateral Stalk Subunit P0), RN7SK (RNA component of 7SK nuclear ribonucleoprotein), SNRPD3 (small nuclear ribonucleoprotein D3 polypeptide), and XRCC5 (X-ray repair cross complementing 5) were used as reference genes; their stability was evaluated by GeNorm algorithm in order to establish the best combination of reference genes [42]. Following this analysis, the geometric mean of SNRPD3 and XRCC5 was taken as reference. The relative expression of MEG3 was calculated with the ΔΔCt method, using the mean ΔCt of all samples as reference sample [43]. The list of primers used is shown in Table S1.

### 4.3. Cell Line and Culture Condition

The human ovarian carcinoma cell lines PEO1 and COV318 (HGSOC cell line) were obtained from the European Collection of Cell Cultures (ECACC, Salisbury, UK) and American Type Culture Collection (ATCC, Milan, Italy), respectively. Susan Horwitz (Albert Einstein Medical College) donated the HEY cell line. The immortalized human ovarian surface epithelial cell line (HOSE, #T1074) was purchased from Applied Biological Materials Inc. (ABM, Richmond, BC, Canada), whereas the immortalized Fallopian tube secretory epithelial cell line FT194 was kindly provided by Dr. MS Zannini, with the authorization of Dr. R Drapkin (Boston, MA, USA). PEO1 and HEY were cultured in RPMI (Roswell Park Memorial Institute) 1640, COV318 in Dulbecco’s modified Eagle’s medium (Sigma-Aldrich, St. Louis, MO, USA), and HOSE in Prigrow I medium (ABM). Medium was supplemented with 10% fetal bovine serum, 1% MEM (Minimal Essential Medium) non-essential amino acid, 1 mM glutamine, and 1% kanamicin. Sodium pyruvate 2 mM was also added to PEO1 medium (Sigma-Aldrich). FT194 was maintained in DMEM-F12 medium (Euroclone, Milan, Italy) containing 2% Ultroser G serum (PALL, Ann Arbor, MI, USA). Cells were grown in a fully humidified atmosphere of 5% CO_2_/95% air, at 37 °C. Cells were routinely tested for mycoplasma (MycoAlert mycoplasma detection kit, LONZA, Rockland, ME, USA) and validated by STR (short tandem repeat) DNA profiling (BMR Genomics srl, Padua, Italy).

### 4.4. MEG3 Overexpression

Full-length MEG3 cDNA (NR_002766) (cDNA, complementary DNA) was cloned into pcDNA 3.1(+) by Genscript (Genscript, Piscataway, NJ, USA), thus obtaining the expression construct pMEG3. PEO1 and HEY cells were transfected with pMEG3 or the empty vector as negative control. Transfectin Lipid Reagent (Bio-Rad, Milan, Italy) was used to transfect plasmids into cell lines, according to the manufacturer’s instructions. The expression level of MEG3 in transfected cells compared to empty vector was measured by RT-qPCR. Cells transiently transfected with pcDNA or pMEG3 were used to perform in vitro assays; stably transfected HEY pcDNA and pMEG3 cells were used for in vivo preclinical study.

### 4.5. Proliferation Assay

For proliferation assay, MEG3-overexpressing cells and corresponding pcDNA control cells (80,000 cells for PEO1 and 50,000 cells for HEY) were seeded in 10 mm plates in complete culture medium. After 24, 48, and 72 h, cells were harvested by trypsinization, and viable cells were counted with NucleoCounter NC-200 (Chemometec, Lillerød, Denmark). The experiment was performed three times.

### 4.6. Clonogenic Assay

For clonogenic assay, MEG3-overexpressing cells and corresponding pcDNA control cells (1000 cells per plate for PEO1 and 100 cells per plate for HEY cell lines) were seeded in 60 mm plates. Ten days post-plating, colonies with more than 50 cells were counted after fixation with ice cold ethanol and staining with crystal violet (0.5% *w/v*). The experiment was performed three times.

### 4.7. Invasion and Migration Assay

For both Transwell migration and invasion assays, 20,000 (HEY) or 80,000 (PEO1) cells were added into the upper chamber of the insert (8 µm pore size; Corning, NY, USA), after 24 h of serum starvation. For invasion assays, the upper chamber of the insert was precoated with Matrigel (Corning, NY, USA). In both assays, the lower chamber contained medium with 10% FBS (Fetal bovine serum) as chemoattractant. After 5 h (HEY) or 24 h (PEO1) of incubation at 37 °C in a 5% CO_2_ atmosphere, cells that did not migrate or invade through the pores were carefully wiped out with cotton wool. Observation times were chosen on the basis of previous data by our group [44]. The inserts were then fixed with ethanol, stained with crystal violet and counted under a microscope. The experiment was performed three times.

### 4.8. Spheroid Cultures

For spheroid cultures, HEY pcDNA and pMEG3 cells were mixed with a solution of VitroGel 3D-RGD (RGD, Arginylglycylaspartic acid) (TheWell, Bioscience, North Brunswick, NJ, USA)/0.5× PBS (Phosphate-buffered saline). Cell suspensions were mixed at a 3:1 ratio (final cell concentration: 2 × 10^5^ cells/mL). Hydrogel/cell mixture was added to well plate and then incubated at room temperature for 20 min. After stabilization, complete cell culture medium was added on the top of the hydrogel. The 3D cell cultures were maintained for 10 days and continuously monitored using a microscope DM IL LED (Leica Microsystems, Milan, Italy).

The number of spheroids was determined by using a Neubauer chamber. For this analysis, spheroids were first recovered from hydrogel by VitroGel Cell Recovery Solution (TheWell, Bioscence), according to the manufacturer’s protocol, and then counted under a microscope. After recovery from hydrogel, spheroids were observed under bright-field microscopy, and Leica Application Suite (LAS) analysis was used to measure their diameters (µm) at day 10. Cell proliferation was assessed after 10 days of culture using the Cell Counting Kit 8 (CCK-8, Sigma-Aldrich), following the manufacturer’s procedure. Optical density (OD) at 450 nm was measured using a multifunction microplate reader (EnSpire, PerkinElmer, Milan, Italy) after incubation for 4 h at 37 °C. For immunofluorescence analysis, spheroids were fixed in 4% paraformaldehyde for 20 min at room temperature, and permeabilized in 0.5% *v/v* Triton X-100 in PBS for 10 min, prior to be blocked with 20% *v/v* serum and 0.1% *v/v* Triton X-100 in PBS for 1 h. Immunofluorescence staining was obtained using anti-β-actin (1:200, AC-15, Sigma-Aldrich), following overnight incubation at 4 °C. After washing, cells were incubated with secondary antibody anti-mouse Alexa Fluor-488 conjugate and DAPI (4′,6-diamidino-2-phenylindole) in the dark for 30 min and 5 min, respectively, at room temperature. Spheroids were recovered from hydrogel by VitroGel Cell Recovery Solution and mounted onto slides that were observed under a fluorescence microscope (Leica Biosystems, Newcastle, UK) using a 100× oil immersion objective.

### 4.9. Animals

Female athymic mice (CAnN.Cg-Foxn1nu/Crl BALB/c nude), 5 weeks old and within a weight range of approximately 18–22 g, were obtained from Charles River (Calco, Lecco, Italy). Animals were housed in a purpose-built facility with a controlled environment, and were maintained in an isolator in which control was set to keep temperature and relative humidity at 26 ± 2 °C and 50%, respectively. Artificial lighting provided a 24 h cycle of 12 h light and 12 h dark. Sterile water and food were supplied ad libitum during the study. Procedures and facilities followed the requirements of Commission Directive 2010/63/UE concerning the protection of animals used for experimental and other scientific purposes. Italian legislation is defined in the Decreto Legislativo No. 26 of 4 March 2014. In addition, the Guidelines for the Welfare and Use of Animals in Cancer Research were followed [45]. The project was approved by the local ethics committee and by the Italian Ministry of Health (Protocol no. 457/2019-PR, 17th June 2019).

### 4.10. Human Tumor Xenograft Growth

On the day of dosing, HEY pcDNA and pMEG3 cells were trypsinised, and a suspension of 2.0 × 10^6^ cells was injected subcutaneously in the right flank of each animal (0.2 mL per mice). There were 12 mice per group. During the study, mice were checked daily for any adverse clinical reactions. Body weight and tumor dimensions were measured three times per week; tumor volume was calculated from two-dimensional measurements (mm), as previously reported [41]: tumor volume = length × width^2^/2. At the end of the study, animals were anesthetized prior to cervical dislocation. All tumors were removed, fixed in 10% formalin, and subsequently dehydrated and blocked in paraffin. The paraffin block was cut into 3 µm sections, fixed on coded slides, and processed for immunohistochemistry (see below).

### 4.11. RNA Extraction from Cell Lines and RT-qPCR Analysis

Total RNA was extracted from cells using RNeasy mini kit (Qiagen) following the manufacturer’s instructions. To determine subcellular localization, nuclear fraction was isolated from cytoplasm using ipotonic buffer (10 mM HEPES, pH 7.5; 10mM KCl; 1.5 MgCl_2_) (HEPES, 4-(2-hydroxyethyl)-1-piperazineethanesulfonic acid) in the presence of RNase and protease inhibitors. Nuclear and cytosolic RNA was then isolated using miRNeasy mini kit (Qiagen), as previously described [46]. For analysis of MEG3 expression in the different cellular models, RNA was reverse-transcribed, pre-amplified, and subjected to RT-qPCR analysis, as detailed above. The geometric mean of SNRPD3 and RPLP0 was taken as reference genes, following GeNorm algorithm [41]; data from total RNA are presented as Log2 fold change (Log2FC) values calculated with the ΔΔCt method, using FT194 or pcDNA as reference sample [43]. Data from fractionated samples are presented as Log2FC values calculated by 2^−ΔCt^ [2^−(Ct NUC − Ct CYT)^] method (ΔCt method) [47] and compared to FT194. Two RNA molecule markers with known nuclear (NEAT1, Nuclear Paraspeckle Assembly Transcript 1) or cytoplasmic (RPPH1, ribonuclease P RNA component H1) enrichment [48] confirmed successful isolation of pure nuclear and cytoplasmic fractions. The list of primers used is shown in Table S1.

### 4.12. Western Blot Analysis

Western blot analysis of total cell lysates was performed as previously described [49]. In detail, total cellular proteins were obtained by lysing the cells with lysis buffer (50 mM Tris-Cl, pH 7.5; 150 mM NaCl; 1% *w/v* Nonidet *p*-40; 0.5% *w/v* sodium deoxycholate, 0.1% *w/v* SDS, 1 mM EDTA) (EDTA, Ethylenediaminetetraacetic acid) in the presence of proteases and phosphatase inhibitors. Equal amounts of protein were separated by SDS polyacrylamide gel electrophoresis, blotted to PVDF (Polyvinylidene fluoride), and transferred using the Trans-Blot Turbo Transfer System (Bio-Rad) with 25 V, 1.0 A, for 30 min. After blocking in 5% non-fat milk (Bio-Rad), membranes were probed with the following primary antibodies: anti-PTEN (1:1000, clone 6H2.1; Agilent Technologies, Santa Clara, CA, USA), anti-p-mTOR (1:1000; Cell Signaling Technology, Danvers, MA), anti-mTOR (1:1000, clone 7C10; Cell Signaling Technology), and anti- β-actin (1:5000, clone AC-15; Sigma-Aldrich), at 4 °C, overnight. After incubation with secondary horseradish peroxidase-conjugated antibodies (Bio-Rad), specific proteins were visualized by the enhanced chemiluminescence system (Amersham Biosciences, Buckinghamshire, UK) using a ChemiDoc XRS+ imaging system (Bio-Rad).

### 4.13. Immunohistochemistry Analysis

Immunohistochemistry analysis was performed on six tumors from pcDNA and six tumors from pMEG3 groups. The process of deparaffinization, rehydration, and epitope retrieval of tissue specimens was performed with low pH Target Retrieval Solution (Agilent Technologies) in DAKO PT Link module (Pre-treatment module, Agilent Technologies). After blocking the endogenous peroxidase activity, the slides were incubated with 20% normal goat serum for 30 min at room temperature and then with Ki67 (ready to use, clone MIB-1; Agilent Technologies) or PTEN (1:100, clone 6H2.1; Agilent Technologies) for 30 min at room temperature. Thereafter, sections were incubated with the secondary antibody, anti-mouse/rabbit EnVision System-HRP (HRP, horseradish peroxidase, Dako, Agilent) (30 min, room temperature). The slides were developed with diaminobenzidine (DAB substrate system, Dako, Agilent), counterstained with Mayer’s haematoxylin, dehydrated in ethanol and xylene, and finally mounted. Staining without primary antibody was used to validate the specificity of the secondary antiserum. Immunohistochemical scoring was determined without knowledge of the treatment groups. For Ki67, the number of positive (brown-stained) cells was determined as a percentage of the total number of cells counted in non-necrotic areas of each section. Scoring of PTEN was evaluated as reported previously [50]. Briefly, the mean percentage of stained cells was categorized as follows: 0 = 0%, 1 = 1–10%, 2 = 11–33%, 3 = 34–66%, and 4 = 67–100%. The staining intensity was also evaluated and graded from 0 to 3, where 0 = no staining, 1 = weak staining, 2 = moderate staining, and 3 = strong staining. The two values obtained were multiplied to calculate a receptor score (maximum value 12).

### 4.14. Statistical Analysis

PFS (progression-free survival) was defined as the interval (in months) between date of diagnosis and date of progression (radiological or clinical assessed) or death, whichever occurred first, or date of last follow-up for patients alive and without progression. The platinum-free interval (PFI) was defined as the time between the last cycle of platinum and evidence of disease progression. OS (overall survival) was defined as the interval between date of diagnosis and date of death (or date of last follow-up for alive patients). Median follow-up was calculated according to the inverted Kaplan–Meier technique [51]. The prognostic effect of the various parameters on clinical outcome (i.e., recurrence or death of disease) was tested by plotting survival curves according to Kaplan–Meier method, and comparing groups using the log rank test, as well as by multivariate analysis using the Cox model. Median gene expression value was used to dichotomize patients into low and high expression groups. Only variables with *p*-value < 0.1 in the univariate analysis were included in multivariate model. Association between MEG3 expression and clinicopathological parameters was evaluated using the Fisher’s exact test. The remaining data were analyzed for homogeneity of variance using an *F* test. If the variances were heterogeneous, log or reciprocal transformations were made in an attempt to stabilize the variances, followed by Student’s *t*-test. If the variances remained heterogeneous, a non-parametric test such as the Mann–Whitney *U* test was used. *p*-values were two-sided, with *p* < 0.05 considered as significant. Statistical analysis was performed using GraphPad Prism 6 (GraphPad Software, Inc. La Jolla, CA, USA) and StatPlus Version v6 (AnalystSoft Inc, Walnut, CA, USA).

## 5. Conclusions

In this study, we addressed the clinical relevance of MEG3 in HGSOC and its implication in cancer biology. We demonstrated that MEG3 is a powerful prognostic biomarker in patients diagnosed with advanced disease. To support and expand these findings, we also proved a close relationship between MEG3 expression levels and modulation of cancer cell related processes in preclinical models of HGSOC, finally providing a new paradigm for developing therapeutic strategies for cancer treatment.

## Figures and Tables

**Table 1:** Clinicopathological characteristics of high-grade serous ovarian cancer (HGSOC) patients.

Characteristics		No. of Patients (%)
All cases		90
Median age, years (range)		56 (25–74)
Presence of ascites		
	No	22 (24.4)
	Yes	68 (75.6)
FIGO Stage		
	IIIC	78 (86.6)
	IVA	6 (6.7)
	IVB	6 (6.7)
Primary treatment strategy		
	PDS	56 (62.2)
	NACT/IDS	34 (37.8)
Residual tumor after surgery		
	0 cm	52 (57.8)
	≤1 cm	9 (10)
	>1 cm	25 (27.8)
	Not available	4 (4.4)
Primary chemotherapy		
	Platinum/paclitaxel	47 (52.2)
	Platinum/paclitaxel/bevacizumab	36 (40)
	Platinum-based	6 (6.7)
	Not available	1 (1.1)
Chemosensitivity		
	Sensitive	56 (62.2)
	Resistant	34 (37.8)
BRCA mutational status		
	BRCA wt	31 (34.5)
	BRCA mut	20 (22.2)
	Not available	39 (43.3)

HGSOC = High-grade serous ovarian cancer. PDS = primary debulking surgery. NACT = neoadjuvant chemotherapy. IDS = interval debulking surgery. BRCA (BReast CAncer gene) wt = BRCA wild type. BRCA mut = BRCA1/BRCA2 mutated.

**Table 2:** Univariate and multivariate analysis of factors affecting PFS in HGSOC patients.

Outcome and Variables		Univariate		Multivariate	
		HR (95%CI)	*p*	HR (95%CI)	*p* *
Age (years)					
	≤56				
	>56	1.2 (0.8–1.9)	0.4	-	-
Ascites					
	No				
	Yes	0.9 (0.6–1.6)	0.8	-	-
FIGO stage					
	IIIC				
	IVA–IVB	1.5 (0.7–3.1)	0.3	-	-
Primary treatment strategy					
	PDS				
	NACT/IDS	1.2 (0.8–2.0)	0.4	-	-
Residual tumor after surgery					
	≤1 cm				
	>1 cm	1.7 (1.0–2.9)	0.07	1.5 (0.9–2.4)	0.1
MEG3					
	Low expression				
	High expression	0.4 (0.2–0.7)	0.0003	0.5 (0.3–0.8)	0.002

PFS = progression-free survival. HGSOC = high-grade serous ovarian cancer. HR = hazard ratio. CI = confidence interval. * *p*-values were derived from the Cox proportional hazards model. PDS = primary debulking surgery. NACT = neoadjuvant chemotherapy. IDS = interval debulking surgery. The median expression value was used as a cut-off value for classification of patients into high and low MEG3 expression. Only variables with *p*-value < 0.1 in the univariate analysis were included in multivariate model. χ^2^ of the model = 12.34, *p*-value = 0.0021.

**Table 3:** Univariate and multivariate analysis of factors affecting OS in HGSOC patients.

Outcome and Variables		Univariate		Multivariate	
		HR (95%CI)	*p*	HR (95%CI)	*p* *
Age (years)					
	≤56				
	>56	1.7 (0.9–3.1)	0.09	1.8 (1.0–3.3)	0.05
Ascites					
	No				
	Yes	0.9 (0.5–1.9)	0.9	-	-
FIGO stage					
	IIIC				
	IVA–IVB	1.9 (0.7–5.2)	0.2	-	-
Primary treatment strategy					
	PDS				
	NACT/IDS	1.2 (0.7–2.3)	0.5	-	-
Residual tumor after surgery					
	≤1 cm				
	>1 cm	1.4 (0.7–2.8)	0.3	-	-
MEG3					
	Low expression				
	High expression	0.5 (0.2–0.8)	0.01	0.4 (0.2–0.8)	0.01

OS = overall survival. HGSOC = high-grade serous ovarian cancer. HR = hazard ratio. CI = confidence interval. * *p*-values were derived from the Cox proportional hazards model. PDS = primary debulking surgery. NACT = neoadjuvant chemotherapy. IDS = interval debulking surgery. The median expression value was used as a cut-off value for classification of patients into high and low MEG3 expression. Only variables with *p*-value < 0.1 in the univariate analysis were included in multivariate model. χ^2^ of the model = 10.45; *p*-value = 0.0054.

**Table 4:** Expression of MEG3 in the overall series.

Characteristics		No. of Patients with Low MEG3	No. of Patients with High MEG3	p *
All cases		45/90	45/90	
Age (years)				
	≤56	29/50	21/50	0.1
	>56	16/40	24/40	
Ascites				
	No	12/22	10/22	0.8
	Yes	33/68	35/68	
FIGO stage				
	IIIC	37/78	41/78	0.4
	IVA–IVB	8/12	4/12	
Primary treatment strategy				
	PDS	31/56	25/56	0.3
	NACT/IDS	14/34	20/34	
Residual tumor after surgery				
	≤1 cm	30/61	31/61	0.8
	>1 cm	11/25	14/25	
	Not available	4/4	0/4	
Chemosensitivity				
	Sensitive	23/56	33/56	0.05
	Resistant	22/34	12/34	
BRCA mutational status				
	BRCA wt	15/31	16/31	0.1
	BRCA mut	4/20	16/20	
	Not available	26/39	13/39	

The median expression value was used as a cut-off value for classification of patients into high and low MEG3 expression. * *p*-values were evaluated using the Fisher’s exact test. PDS = primary debulking surgery. NACT = neoadjuvant chemotherapy. IDS = interval debulking surgery. BRCA wt = BRCA wild type. BRCA mut = BRCA1/BRCA2 mutated.

## Supplementary Materials

The following are available online at https://www.mdpi.com/2072-6694/12/4/966/s1, Table S1: Delta gene assays for long non-coding RNA (lncRNA) expression, Figure S1: Uncropped blots of Figure 4E, Table S2: Densitometric analysis of Figure 4E.
