# Supplementary material for: Clinical Value of lncRNA MEG3 in High-Grade Serous Ovarian Cancer
Source: Cancers (Basel). 2020 Apr 14;12(4):966. doi: 10.3390/cancers12040966 (PMC7226118; doi:10.3390/cancers12040966)
Supplement: Supplementary file 1 [file cancers-12-00966-s001.pdf]

Supplementary Materials: Clinical Value of lncRNA MEG3 in High-Grade Serous Ovarian Cancer

Marianna Buttarelli, Marta De Donato, Giuseppina Raspaglio, Gabriele Babini, Alessandra Ciucci, Enrica Martinelli, Pina Baccaro, Tina Pasciuto, Anna Fagotti, Giovanni Scambia and Daniela Gallo

Table S1. Delta Gene assays for lncRNA expression.

GENE ID	Gene Symbol	Primer Forward	Primer Reverse	Amplicon Length (bp)
2597	GAPDH	GAACGGGAAGCTTGTCATCAA	ATCGCCCCACTTGATTTTGG	79
3188	HNRNPH2	CGCTATAGCCGTTTGAGGGAA	GGCAAGTTTGGCTCAATGCA	84
55384	MEG3	CCGGACCCAAGTCTTCTTCC	CCTTCCCACGTAGGCATCC	86
283131	NEAT1	GTGCTTGGAACCTTGCTTCAA	CAATCTGCGTTGTGGCATCA	73
6175	RPLP0	GCGACCTGGAAGTCCAAC	CACATTGTCTGCTCCCACAA	87
85495	RPPH1	CGGAGGGAAGCTCATCAGTG	TCAGGGAGAGCCCTGTTAGG	117
125050	RN7SK	ATAGAGGAGGACCGGTCTTCGG	ACTGCCACATGCAGCGCCTC	158
6634	SNRPD3	GCTCATTGAAGCAGAGGACAA	CCACTCGGCCATCTCTGTA	74
7520	XRCC5	GCGTGGCTTTTCCTCATATCA	GTATTGCCGCAAGTCTTCCA	83

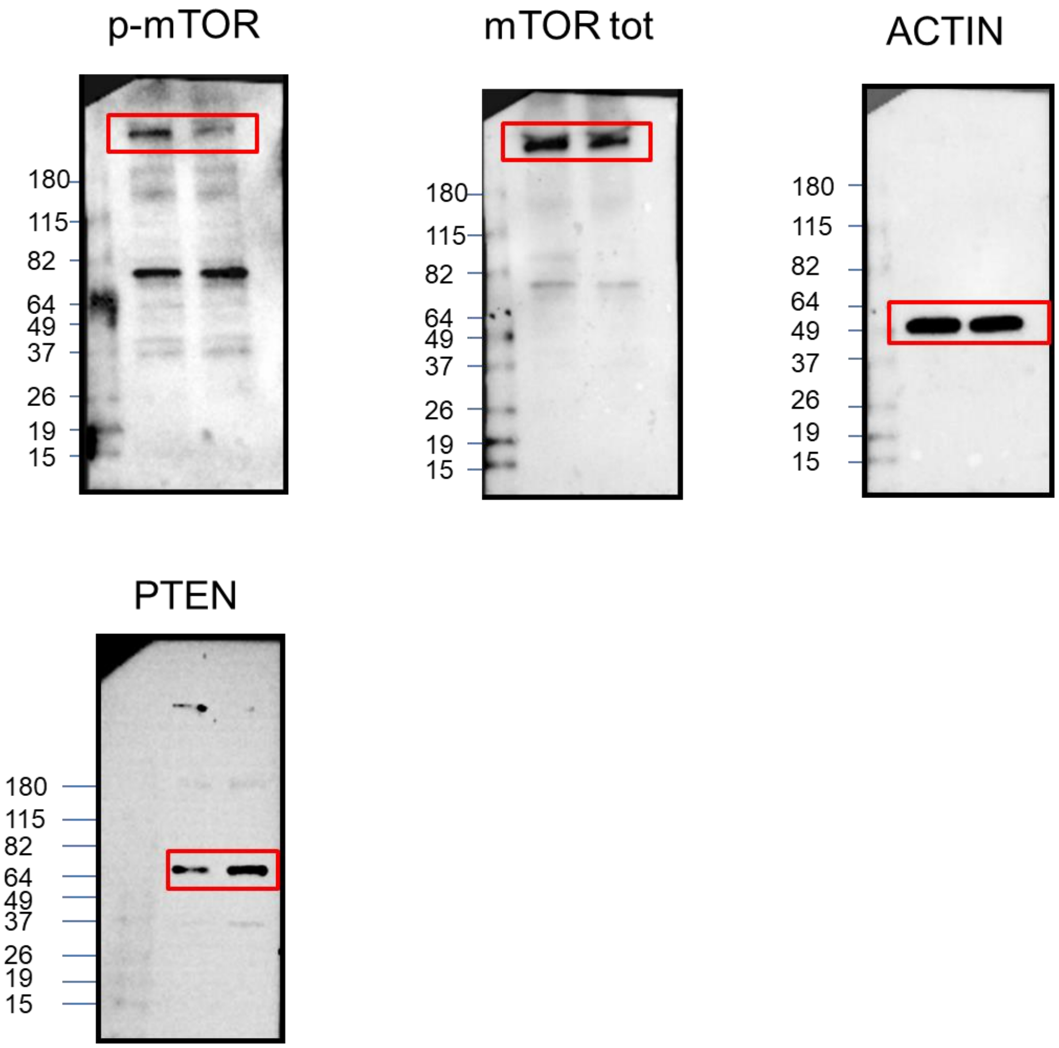

Figure S1. Uncropped blots of Figure 4E. MARKER: BenchMark Pre-Stained Protein Ladder (Invitrogen, Thermofisher Scientific, Waltham, MA, USA).

Table S2. Densitometric analysis of Figure 4E.

p-mTOR	Adj. Total Lane Vol. (Int)	Total Lane Vol. (Int)	Bkgd. Vol. (Int)
Hey pCDNA	804.275	6.284.875	5.480.600
Hey pMEG3	453.200	5.961.575	5.508.375
mTOR tot	Adj. Total Lane Vol. (Int)	Total Lane Vol. (Int)	Bkgd. Vol. (Int)
Hey pCDNA	10.259.810	17.442.684	7.182.874
Hey pMEG3	7.873.068	15.562.406	7.689.338
PTEN	Adj. Total Lane Vol. (Int)	Total Lane Vol. (Int)	Bkgd. Vol. (Int)
Hey pCDNA	2.260.286	4.948.054	2.687.768
Hey pMEG3	3.326.292	6.355.880	3.029.588
Actin	Adj. Total Lane Vol. (Int)	Total Lane Vol. (Int)	Bkgd. Vol. (Int)
Hey pCDNA	4.215.662	4.895.198	679.536
Hey pMEG3	3.973.917	4.715.943	742.026

Acquisition Information: Imager ChemiDoc™ XRS+ Software Version 6.0.1.34 Application Chemi.

© 2020 by the authors. Licensee MDPI, Basel, Switzerland. This article is an open access article distributed under the terms and conditions of the Creative Commons Attribution (CC BY) license (http://creativecommons.org/licenses/by/4.0/).
